# Stages of hyperglycemia and common mental disorders in adults - The Brazilian Study of Adult Health (ELSA-Brasil): Estágios de hiperglicemia e transtornos mentais comuns em adultos - Estudo Longitudinal de Saúde do Adulto (ELSA-Brasil)

**DOI:** 10.1590/1516-3180.2016.0163030716

**Published:** 2016-09-26

**Authors:** Marina Bessel, Álvaro Vigo, Andréa Poyastro, Maria Angélica Nunes, Bruce Bartholow Duncan, Maria Inês Schmidt

**Affiliations:** I MSc. Postgraduate Program on Epidemiology, Universidade Federal do Rio Grande do Sul (UFRGS), Porto Alegre, RS, Brazil.; II PhD. Associate Professor, Department of Statistics and Postgraduate Program on Epidemiology, School of Medicine, Universidade Federal do Rio Grande do Sul (UFRGS), Porto Alegre, RS, Brazil.; III MD, PhD. Postdoctoral Fellow, Postgraduate Program on Epidemiology, School of Medicine, Universidade Federal do Rio Grande do Sul (UFRGS), Porto Alegre, RS, Brazil.; IV MD, PhD. Professor in the Postgraduate Program on Epidemiology, School of Medicine, Universidade Federal do Rio Grande do Sul (UFRGS), Porto Alegre, RS, Brazil.; V MD, PhD. Professor, Department of Social Medicine and Postgraduate Program on Epidemiology, School of Medicine, Universidade Federal do Rio Grande do Sul (UFRGS), Porto Alegre, RS, Brazil.; VI MD, PhD. Professor, Department of Social Medicine and Postgraduate Program on Epidemiology, School of Medicine, Universidade Federal do Rio Grande do Sul (UFRGS), Porto Alegre, RS, Brazil.

**Keywords:** Diabetes mellitus, Prediabetic state, Hemoglobin A, glycosylated, Depressive disorder, Mental disorders, Diabetes mellitus, Estado pré-diabético, Hemoglobina A glicosilada, Transtorno depressivo, Transtornos mentais

## Abstract

**CONTEXT AND OBJECTIVE:**

Diabetes mellitus and depressive disorders frequently coexist. However, this relationship has been little evaluated across stages of hyperglycemia and for a broad range of common mental disorders (CMDs). The objective here was to investigate the association between CMDs and stages of glycemia.

**DESIGN AND SETTING:**

Cross-sectional study conducted among civil servants aged 35-74 years participating in the ELSA-Brasil cohort.

**METHODS:**

CMDs were classified using the Clinical Interview Schedule - Revised (CIS-R). Glycemia was classified in stages as normal, intermediate hyperglycemia, newly classified diabetes or previously known diabetes, based on oral glucose tolerance testing, glycated hemoglobin (HbA1c), self-reported diabetes and medication use. Blood glucose control was assessed according to HbA1c.

**RESULTS:**

CMDs were most prevalent in individuals with previously known diabetes. After adjustments, associations weakened considerably and remained significant only for those with a CIS-R score ≥ 12 (prevalence ratio, PR: 1.15; 95% confidence interval, CI: 1.03-1.29). Intermediate hyperglycemia did not show any association with CMDs. For individuals with previously known diabetes and newly classified diabetes, for every 1% increase in HbA1c, the prevalence of depressive disorders became, respectively, 12% and 23% greater (PR: 1.12; 95% CI: 1.00-1.26; and PR: 1.23; 95% CI: 1.04-1.44).

**CONCLUSION:**

Individuals with previously known diabetes had higher CIS-R scores. Among all individuals with diabetes, worse blood glucose control was correlated with depressive disorder. No relationship between intermediate hyperglycemia and CMDs was observed, thus suggesting that causal processes relating to CMDs, if present, must act more proximally to diabetes onset.

## RESUMO

**CONTEXTO E OBJETIVO:**

Diabetes *mellitus* e transtornos depressivos frequentemente coexistem. No entanto, essa relação tem sido pouco avaliada nos estágios hiperglicêmicos e em uma amplitude maior de transtornos mentais comuns (TMCs). O objetivo foi investigar a associação entre TMCs e estágios de glicemia.

**TIPO DE ESTUDO E LOCAL:**

Estudo transversal realizado com funcionários públicos com idade entre 35-74 anos participantes da coorte ELSA-Brasil.

**MÉTODOS:**

TMCs foram classificados usando o instrumento Clinical Interview Schedule - Revised (CIS-R). Para a classificação dos estágios de glicemia, foi utilizado o teste de tolerância a glicose, hemoglobina glicada (HbA1c), relato pessoal de diabetes e uso de medicamentos. A glicemia foi categorizada como: normal, hiperglicemia intermediária, classificação nova de diabetes, e diabetes prévio. Controle glicêmico foi avaliado pela HbA1c.

**RESULTADOS:**

TMCs foram mais prevalentes nos pacientes com diabetes prévio. Após ajustes, as associações foram consideravelmente enfraquecidas, permanecendo significativas somente para aqueles com escore do CIS-R ≥ 12 (razão de prevalência, RP: 1,15; intervalo de confiança de 95%, IC: 1,03-1,29). Hiperglicemia intermediária não teve associação com CMDs. Para aqueles com diabetes prévio e classificação nova de diabetes, para cada aumento de 1% na HbA1c, a prevalência de transtorno depressivo foi, respectivamente, 12% e 23% maior (RP: 1,12; IC: 1,00-1,26 e RP: 1,23; IC: 1,04-1,44).

**CONCLUSÃO:**

Aqueles com diabetes prévio tiveram escore do CIS-R mais elevado. Entre todos com diabetes, o controle glicêmico pior foi relacionado ao transtorno depressivo. Não foi observada relação entre hiperglicemia intermediária e TMCs, sugerindo que a relação causal relacionada aos TMCs, se presente, deve agir de forma mais próxima ao início de diabetes.

## INTRODUCTION

Chronic non-communicable diseases (NCDs) are responsible for two thirds of the total number of deaths worldwide.^1^ Four diseases, including diabetes mellitus, account for most of these deaths.^2^ The prevalence of diabetes is increasing at epidemic proportions, creating a major challenge to healthcare systems worldwide.^3^

NCDs also generate high disease burden and among these, diabetes and neuropsychiatric disorders, especially depressive and anxiety disorders, have taken on utmost importance.^3^^,^^4^ Depressive disorders occur more frequently among individuals with diabetes and have in fact been considered to be both the cause and the consequence of diabetes.^5^^,^^6^ On the one hand, receiving a diagnosis of diabetes and needing to cope with this chronic disease that has high morbidity and complex management could, in itself, lead to development of depressive symptoms.^7^ On the other hand, factors relating to depressive symptoms, such as obesity, low physical activity or hypercaloric diet, as well as activation of neuroendocrine and inflammatory pathways relating to depression, could also induce insulin resistance and lead to type 2 diabetes. The findings from two meta-analyses on longitudinal studies support each of these directions: a 37% higher risk (relative risk, RR = 1.37; confidence interval, 95% CI: 1.14-1.63) of developing diabetes was seen among adults with depression than among those without depression;^8^ and a 24% higher risk (RR = 1.24; CI 95%: 1.09-1.40) of developing depression among individuals with diabetes than among those without diabetes.^9^

The relationship between depression and milder states of hyperglycemia has been less investigated. Moreover, to our knowledge, glycated hemoglobin, which has been widely used to asses glycemic control among individuals with diabetes^10^ and has more recently been introduced as a tool to diagnose diabetes and milder states of hyperglycemia, has not been used to assess this relationship across the full range of hyperglycemia, from normality to diabetes.

The Brazilian Longitudinal Study of Adult Health (Estudo Longitudinal de Saúde do Adulto, ELSA-Brasil) was conducted on a sample of 15,105 adults who were assessed using the Clinical Interview Schedule - Revised (CIS-R). This tool makes it possible to diagnose distinct common mental disorders (CMDs) and provides full accounting of hyperglycemia. Thus, ELSA-Brasil offers an excellent opportunity to carry out these broad evaluations.

## OBJECTIVE

The purpose of this study was to investigate associations between stages of glycemia (normality, intermediate states, a single laboratory-based diagnosis of diabetes and a clinical diagnosis of diabetes) and CMDs among ELSA-Brasil participants. We also investigated associations between glycated hemoglobin levels and common mental disorders across the various stages of hyperglycemia. 

## METHODS

### Design and study sample

ELSA-Brasil was a prospective cohort study designed to identify risk factors for diabetes and cardiovascular disease. The details of the study methodology, including design and eligibility criteria, were described previously.^11^^,^^12^ The cohort comprised 15,105 civil servants who were 35-74 years old at the baseline (2008-2010) and were sampled from universities or research institutes located in six Brazilian state capitals (São Paulo, Belo Horizonte, Porto Alegre, Salvador, Rio de Janeiro and Vitoria). All active or retired employees of the institutions involved, aged 35-74 years, were eligible for the study. The ethics committee of each institution approved the research protocol, and volunteers gave written consent to participate.

### Analytical sample

Out of the 15,105 participants, we excluded 658 participants for whom values relating to the main outcomes, covariates or variables needed to classify stages of diabetes were missing: 10 lacking information on CIS-R, 20 on glycated hemoglobin, 396 on covariates and 232 on self-reported diabetes, treatment for diabetes (medication or diet) or measurements of fasting or two-hour plasma glucose. Thus, our analytical sample was formed by 14,447 participants.

### Common mental disorders (CMDs)

The CIS-R (Clinical Interview Schedule - Revised) was used to measure occurrences of actual psychiatric morbidity (depression and anxiety symptoms).^13^ The complete CIS-R version includes 14 sections covering symptoms of CMDs that are present at a level that causes distress and interference with daily activities.

Each section begins with a number of mandatory filter questions that establish whether a particular symptom was present during the past month. The presence of a positive symptom leads to a more detailed assessment of the specific symptom over the past week (frequency, duration, severity and time since onset), in order to determine a score for each section.^14^ The CIS-R psychiatric morbidity can be assessed by adding up all 14 symptoms. A CIS-R score ≥ 12 was used to indicate an elevated score.^13^^,^^15^^,^^16^

Additionally, diagnoses of specific disorders were obtained by applying algorithms based on the ICD-10 diagnostic criteria (World Health Organization)^14^ and examining the responses to various sections of the CIS-R. The CIS-R allows five diagnostic categories: generalized anxiety disorder, depressive episode, all phobias (agoraphobia, social phobia and simple phobia), obsessive-compulsive disorder and panic disorder. Also, a diagnosis of mixed anxiety and depression disorder (MADD) can be made in the presence of CIS-R ≥ 12 that does not fulfill the criteria for any of these five ICD-10 diagnostic categories. We grouped these disorders into three major groups: all types and severities of depressive episodes, all anxiety disorders (AD; comprising general anxiety disorder, panic disorder, social anxiety disorder, phobias and obsessive-compulsive disorders) and MADD.

### Stages of hyperglycemia and glucose control 

Presence of diabetes mellitus was ascertained by means of self-reporting of diagnosis and medication use, fasting glucose measurement and an oral glucose tolerance test (OGTT). Individuals were classified as presenting previously known diabetes if they answered "yes" to either of the following questions: "Have you previously been told by a physician that you had/have diabetes (sugar in the blood)?" or "Have you used medication for diabetes in the past two weeks?"; or if they reported any changes to their dietary habits due to diabetes. The remaining participants were classified according to laboratory measurements. Participants with fasting glucose ≥ 126 mg/dl, two-hour plasma glucose ≥ 200 mg/dl or HbA1c ≥ 7% were classified as presenting new-onset diabetes. Those with impaired fasting glucose (fasting glucose ≥ 110 mg/dl and < 126 mg/dl) or impaired glucose tolerance (two-hour plasma glucose ≥ 140 mg/dl and < 200 mg/dl) were classified as having intermediate hyperglycemia. Those not meeting the above criteria were classified as having normal glycemia.

The stages of glycemia were classified hierarchically as four distinct groups: previously known diabetes (n = 1096), newly classified diabetes based only on laboratory measurements (n = 1336), intermediate hyperglycemia (n = 4841), and normal glycemia (n = 7174).

Glucose control was assessed using glycated hemoglobin (HbA1c), which represents the average blood glucose over the past 90 days. HbA1c was measured by means of high-pressure liquid chromatography (Bio-Rad Laboratories, Hercules, California, USA), using a method certified by the National Glycohemoglobin Standardization Program.

### Covariates

Sociodemographic characteristics and behavioral risk factors were obtained through structured questionnaires. Anthropometry was obtained using standardized protocols. The body mass index (BMI) was defined as weight (kg) divided by height squared (m^2)^ and the waist-hip ratio as waist circumference divided by hip circumference.^6^^,^^17^

### Statistical analysis

Associations between common mental disorders and hyperglycemic stages were estimated using Poisson regression models with robust variance, separately for the four dichotomous outcomes: CIS-R score ≥ 12, depressive disorders (DD), anxiety disorder (AD) and mixed anxiety and depression disorder (MADD), using the normal glycemia participants as the reference group.

The same models were used to estimate associations between the abovementioned dichotomous outcomes and glucose control defined by HbA1C, separately for each stage of hyperglycemia, except for normal glycemia.

All the analyses were performed using the Statistical Analysis System (SAS) software, version 9.4, taking the significance level to be 5%.

## RESULTS

Table 1 describes the study participants, overall and stratified according to glucose status. Women predominated (54.1%), as did Caucasians (52.2%), individuals between 45 and 64 years of age (67.5%), married individuals (66.3%) and individuals who reported only doing limited physical exercise during their leisure time (76.8%). The prevalence of common mental disorders (CIS-R ≥ 12) was 26.7% (95% CI: 26.0-27.4), such that 4.3% (95% CI: 3.9-4.6) of the participants presented a depressive disorder, 16.1% (95% CI: 15.5-16.7) an anxiety disorder and 12.7% (95% CI: 12.2-13.2) a mixed anxiety and depression disorder.

**Table 1:**
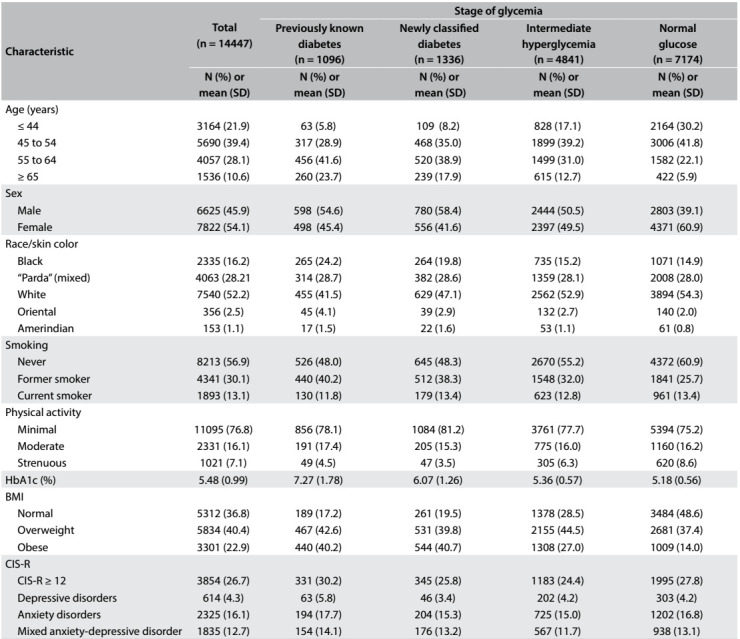
Participants' characteristics: overall and according to stage of glycemia. Longitudinal Study of Adult Health (ELSA-Brasil), 2008-2010

SD = standard deviation; HbA1c = hemoglobin A1c; BMI = body mass index; CIS-R = Clinical Interview Schedule - Revised.

Table 2 presents prevalence ratios (PR) for the presence of common mental disorders at different stages of hyperglycemia, with normal glucose as the reference category. In minimally adjusted models, individuals with previously known diabetes had greater prevalence of all common mental disorders. Lesser differences in prevalence were observed for individuals classified as diabetic based only on laboratory values. Regarding depressive and anxiety disorders, the differences were not statistically significant. After adjustments had been made for physical activity and also especially for obesity indices, the associations weakened considerably. The only remaining association was for individuals with previously known diabetes and, among them, only for elevated CIS-R scores (prevalence ratio, PR = 1.15; 95% CI: 1.03-1.29). Intermediate states of hyperglycemia were never associated with common mental disorders, even in the minimally adjusted analyses.

**Table 2:**
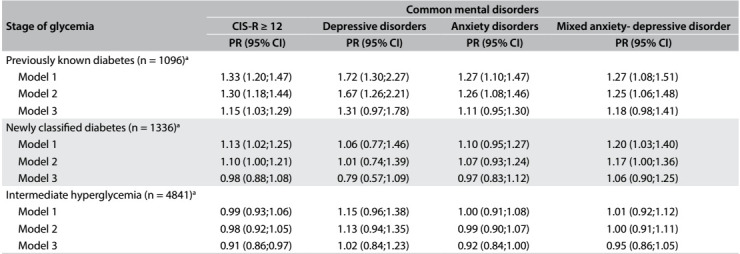
Association of common mental disorders with stage of glycemia. Longitudinal Study of Adult Health (ELSA-Brasil), 2008-2010

PR = prevalence ratio estimated through Poisson regression with robust variance; ^a^normal glycemia participants (N = 7174) are the reference group. Model 1: adjusted for sex, age, race, marital status and smoking; Model 2: adjusted additionally for physical activity; Model 3: adjusted additionally for body mass index and waist-hip ratio.

Table 3 presents the associations with a somewhat different approach. Within each stage of glycemia, the association with mental disorders is expressed in terms of a 1% difference in glycated hemoglobin. Here, greater prevalences were observed primarily for individuals with diabetes that was determined through laboratory abnormalities (previously unknown diabetes), and the associations were strongest for depressive disorders (PR = 1.23; 95% CI: 1.04-1.44, in the best-fit model). Again, no association was found with regard to individuals with intermediate hyperglycemia, except for borderline significantly higher CIS-R scores in less adjusted models.

**Table 3:**
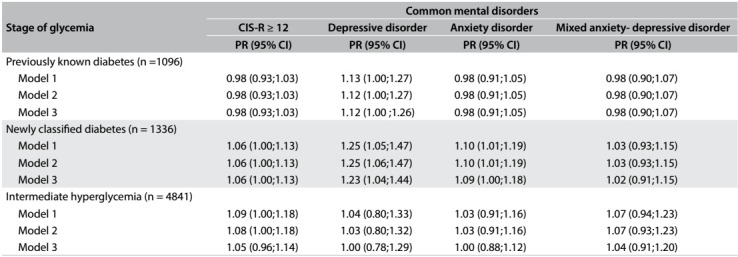
Association^a^ between glycated hemoglobin and presence of common mental disorders at different stages of glycemia. Longitudinal Study of Adult Health (ELSA-Brasil), 2008-2010

PR = prevalence ratio estimated through Poisson regression with robust variance; ^a^expressed for a 1% difference in glycated hemoglobin. Model 1: adjusted for sex, age, race, marital status and smoking; Model 2: adjusted additionally for physical activity; Model 3: adjusted additionally for body mass index and waist-hip ratio.

## DISCUSSION

Consistent with previous studies,^9^ we found that all of the common mental disorders investigated were more prevalent (27% to 72%) among individuals with previously known diabetes than among individuals with normal glycemia, in minimally adjusted models. Das-Munshi et al., using the same instrument (CIS-R) for classifying mental disorders, found a 50% higher prevalence (odds ratio, OR = 1.5; 95% CI: 1.1-2.2) of common mental disorders and a 70% higher prevalence (OR = 1.7; 95% CI: 1.1-2.6) of mixed anxiety and depression disorders among individuals with previously known diabetes, in similarly minimally adjusted models.^17^ We also found that among individuals with previously known diabetes, having a higher level of glycated hemoglobin was associated with a slightly, though statistically significantly greater presence of depressive disorders (12% for every 1% increase in HbA1c), which is consistent with previous reports.^18^

More frequent presence of mental disorders among individuals with diabetes may be explained by the higher burden resulting from having to deal with a chronic disease that presents multiple acute and chronic complications and for which the treatment is long-term and complex. Additionally, two acute complications, hypoglycemia and hyperglycemia, may activate the hypothalamic/pituitary/adrenal axis and thus lead to depression.^19^ Altered neurotrophins, presence of inflammatory mediators and reduced white mass are also related to depression among individuals with diabetes.^20^

Interestingly, among individuals who did not know that their glucose levels had reached the criterion for defining diabetes, we found a 23% greater prevalence (PR = 1.23; 95% CI: 1.04-1.44) of depressive disorders with a 1% increase in glycated hemoglobin. This somewhat larger association is unlikely to result from the burden of treating diabetes and its complications, since the affected individuals were unaware of their high glucose status and were also more likely to be in earlier stages of the disease and, as such, less likely to suffer from its complications. However, it is possible that symptomatic hyperglycemia was present in some of them, and this may have been causing depressive symptoms through activation of the hypothalamic/pituitary/adrenal axis, or through production of symptoms such as fatigue and insomnia, which are among the criteria used for determining the presence of depression.

Another possible explanation for the associations found is that hyperglycemia results, in part, from a complex inflammatory/metabolic condition that may also produce psychoneuroendocrine comorbidity.^21^^,^^22^ In fact, as has been demonstrated with various inflammation markers,^23^^,^^24^^,^^25^^,^^26^^,^^27^ mild chronic inflammation precedes and predicts the development of diabetes in adults.

On the other hand, having a depressive disorder has been shown to predict poorer glycemic control among individuals with type 2 diabetes.^28^ However, the fact that in our analyses, intermediate hyperglycemia was never associated with common mental disorders argues somewhat against this directionality. Additionally, we did not find any association between glycated hemoglobin and common mental disorders among individuals with intermediate hyperglycemia.

Given the possible bidirectionality of the associations between diabetes and common mental disorders, caution must be maintained in interpreting causality. Golden et al.^5^ examined both directions of this association and found that there was a modest association between baseline depressive symptoms and the incidence of type 2 diabetes that was partially explained by lifestyle factors. They also found that individuals with type 2 diabetes that was under treatment more frequently developed depressive symptoms, and that this association was not substantively affected by adjustment for potential confounding or mediating factors.

Our study has some notable strengths. To our knowledge, it is the most comprehensive cross-sectional study to date, given that we investigated both a large spectrum of hyperglycemic stages and several common mental disorders. We undertook a full assessment of diabetes, encompassing participants' reports on diagnoses and medication use, an oral glucose tolerance test and measurements on glycated hemoglobin. We also carefully assessed common mental disorders. Our relatively large sample size enabled adjustment for several potential confounding factors, thus demonstrating associations independent of smoking, marital status, age, sex, race and, to some extent, sedentary behavior and obesity.

Some limitations of our study need to be borne in mind. First, the cross-sectional nature of our findings limits interpretation of the possible direction of the associations presented. Second, we were unable to distinguish types of diabetes, although it is likely that the vast majority of the subjects had type 2 diabetes. Third, our determination of the presence of mental disorders did not evaluate their recurrence or chronicity.^17^ Fourth, we did not consider the role of antidiabetic or antidepressive medication in the associations. It is possible, for instance, that metformin use might decrease the risk of depression, which could explain the slightly lower prevalence ratio for depression seen among treated cases than among the newly diagnosed cases.^20^^,^^29^^,^^30^ Finally, modeling these associations is tricky, given their probable bidirectionality, their multiple common causes and the possibility that various covariates may act as confounders and mediators. In fact, we found that after adjustments for lower physical activity levels and greater obesity indices, both of which may be a cause or a consequence of diabetes or its treatment, the associations between common mental disorders and known diabetes weakened considerably, and remained statistically significant only for individuals who presented an elevated CIS-R score (PR = 1.15; 95% CI: 1.03-1.29).

Our findings strengthen the previous results in the literature, through documenting the coexistence of diabetes and common mental disorders, especially depression. Adequate glucose control, in addition to preventing diabetic complications, may also prevent depressive disorders. If so, additional investigations will be required in order to determine whether this prevention results from glucose control *per se* or whether it arises from the ability to cope with the disease, for instance in terms of self-efficacy in managing diabetes and other conditions such as weight control and physical activity.^2^

## CONCLUSION

Individuals with previously known diabetes had higher CIS-R scores. Among all individuals with diabetes, worse blood glucose control was correlated with depressive disorder. No relationship between intermediate hyperglycemia and CMDs was observed, thus suggesting that causal processes relating to CMDs, if present, must act more proximally to diabetes onset.

## References

[1] World Health Organization (WHO) (2011). Noncommunicable diseases country profiles 2011.

[2] Schmidt MI, Duncan BB, Azevedo e Silva G (2011). Chronic non-communicable diseases in Brazil burden and current challenges. Lancet.

[3] International Diabetes Federation IDF diabetes atlas.

[4] Institute for Health Metrics and Evaluation.

[5] Golden SH, Lazo M, Carnethon M (2008). Examining a bidirectional association between depressive symptoms and diabetes. JAMA.

[6] Kivimaki M, Tabak AG, Batty GD (2009). Hyperglycemia, type 2 diabetes, and depressive symptoms the British Whitehall II study. Diabetes Care.

[7] Golden SH, Lee HB, Schreiner PJ (2007). Depression and type 2 diabetes mellitus the multiethnic study of atherosclerosis. Psychosom Med.

[8] Knol MJ, Twisk JW, Beekman AT (2006). Depression as a risk factor for the onset of type 2 diabetes mellitus A meta-analysis. Diabetologia.

[9] Nouwen A, Winkley K, Twisk J (2010). Type 2 diabetes mellitus as a risk factor for the onset of depression a systematic review and meta-analysis. Diabetologia.

[10] Sumita NM, Andriolo A (2008). Importância da hemoglobina glicada no controle do diabetes mellitus e na avaliação de risco das complicações crônicas (revisão) [Glycohemoglobin importance in the diabetes mellitus control and in the risk evaluation of chronic complications: (review)]. J Bras Patol Med Lab.

[11] Schmidt MI, Duncan BB, Mill JG (2015). Cohort Profile Longitudinal Study of Adult Health (ELSA-Brasil). Int J Epidemiol.

[12] Aquino EM, Barreto SM, Bensenor IM (2012). Brazilian Longitudinal Study of Adult Health (ELSA-Brasil) objectives and design. Am J Epidemiol.

[13] Lewis G, Pelosi AJ, Araya R, Dunn G (1992). Measuring psychiatric disorder in the community a standardized assessment for use by lay interviewers. Psychol Med.

[14] World Health Organization International Classification of Diseases and Related Health Problemas.

[15] Skapinakis P, Anagnostopoulos F, Bellos S (2011). An empirical investigation of the structure of anxiety and depressive symptoms in late adolescence cross-sectional study using the Greek version of the revised Clinical Interview Schedule. Psychiatry Res.

[16] Office for National Statistics (2000). Psychiatric Morbidity Among Adults Living In Private Households.

[17] Das-Munshi J, Stewart R, Ismail K (2007). Diabetes, common mental disorders, and disability findings from the UK National Psychiatric Morbidity Survey. Psychosom Med.

[18] Lustman PJ, Anderson RJ, Freedland KE (2000). Depression and poor glycemic control a meta-analytic review of the literature. Diabetes Care.

[19] Knol MJ, Heerdink ER, Egberts ACG (2007). Depressive symptoms in subjects with diagnosed and undiagnosed type 2 diabetes. Psychosom Med.

[20] Stuart MJ, Baune BT (2012). Depression and type 2 diabetes inflammatory mechanisms of a psychoneuroendocrine co-morbidity. Neurosci Biobehav Rev.

[21] Schmidt MI, Duncan BB (2003). Diabesity an inflammatory metabolic condition. Clin Chem Lab Med.

[22] Duncan BB, Schmidt MI (2001). Chronic activation of the innate immune system may underlie the metabolic syndrome. Sao Paulo Med J.

[23] Schmidt MI, Duncan BB, Sharrett AR (1999). Markers of inflammation and prediction of diabetes mellitus in adults (Atherosclerosis Risk in Communities study) a cohort study. Lancet.

[24] Duncan BB, Schmidt MI, Pankow JS (2003). Low-grade systemic inflammation and the development of type 2 diabetes the atherosclerosis risk in communities study. Diabetes.

[25] Duncan BB, Schmidt MI, Pankow JS (2004). Adiponectin and the development of type 2 diabetes the atherosclerosis risk in communities study. Diabetes.

[26] Schmidt MI, Duncan BB, Vigo A (2006). Leptin and incident type 2 diabetes risk or protection?. Diabetologia.

[27] Luft VC, Schmidt MI, Pankow JS (2013). Chronic inflammation role in the obesity-diabetes association a case-cohort study. Diabetol Metab Syndr.

[28] Papelbaum M, Moreira RO, Coutinho W (2011). Depression, glycemic control and type 2 diabetes. Diabetol Metab Syndr.

[29] Diabetes.co.uk Common diabetes drugs could combat depression.

[30] Wahlqvist ML, Lee MS, Chuang SY (2012). Increased risk of affective disorders in type 2 diabetes is minimized by sulfonylurea and metformin combination a population-based cohort study. BMC Med.

